# Changes in Receiving Preventive Care Services Among US Adults With Diabetes, 1997-2007

**Published:** 2010-04-15

**Authors:** Carmen D. Harris, Liping Pan, Qaiser Mukhtar

**Affiliations:** Centers for Disease Control and Prevention; Centers for Disease Control and Prevention, Atlanta, Georgia; Centers for Disease Control and Prevention, Atlanta, Georgia

## Abstract

**Introduction:**

Diabetes is a chronic disease that requires complex continuing medical care and patient self-management to reduce the risk of long-term complications. Receipt of multiple recommended preventive care services can prevent or delay diabetes-related complications such as blindness and lower-extremity amputations.

**Methods:**

We analyzed 1997 and 2007 Behavioral Risk Factor Surveillance System data to examine change in rates of adults with diabetes receiving 4 essential preventive care services (influenza and pneumococcal vaccinations and annual foot and eye examinations).

**Results:**

The overall age-adjusted rate of receiving all 4 of the preventive care services was 10% in 1997 but increased to 20% in 2007. Rates for receiving all 4 services increased significantly in all demographic subgroups except Hispanics.

**Conclusion:**

Use of preventive care services is increasing, but most US adults with diabetes do not meet recommendations, and the problem is particularly pronounced among Hispanics. The need to receive preventive care services should continue to be emphasized in clinical and community settings to increase the percentage of adults with diabetes who receive them.

## Introduction

In 2007, 23.6 million people, or nearly 8% of the US population, were estimated to have diabetes (1). Diabetes is a chronic disease that requires complex, continuing medical care and patient self-management to reduce the risk of long-term complications (2). These complications include cardiovascular disease, kidney disease, neuropathy, blindness, and lower-extremity amputation (3). To reduce the risk of these complications, the American Diabetes Association (ADA) recommends multiple preventive care services, such as self-monitoring blood glucose levels, annual foot and dilated eye examinations, and vaccinations as standards of continuing care for those diagnosed with diabetes (2). Similarly, *Healthy People 2010* includes objectives to improve the proportion of adults with diabetes who receive annual foot and eye examinations, annual influenza vaccination, and at least 1 pneumococcal vaccination in their lifetimes (4).

Only 4 of 10 US adults with diabetes report receiving multiple preventive care services (5). Past studies have also found racial and ethnic disparities in use of preventive care services among adults with diabetes (6-8). To assess changes in rates of receiving comprehensive preventive care among US adults with diabetes, we analyzed Behavioral Risk Factor Surveillance System (BRFSS) data from 1997 through 2007 on receipt of influenza and pneumococcal vaccinations and annual foot and eye examinations.

## Methods

BRFSS is an ongoing state-based, random-digit-dialed telephone survey of the noninstitutionalized US civilian population aged 18 years or older. The survey is conducted in all 50 states, the District of Columbia, and 3 US territories. We defined people with diabetes as respondents who answered yes to the question, "Have you ever been told by a doctor that you have diabetes?" We excluded women who reported having diabetes only during pregnancy and respondents who reported prediabetes or borderline diabetes. Respondents who answered at least 1 to the question, "About how many times in the past 12 months has a health professional checked your feet for any sores or irritations?" were defined as having received a foot examination. Respondents who answered "within the past month" or "within the past year" to the question "When was the last time you had your eyes examined by any doctor or eye care provider?" were defined as having received an eye examination. Respondents who answered yes to the question "During the past 12 months, have you had a flu shot?" were defined as having had an influenza vaccination. Respondents who answered yes to the question "Have you ever had a pneumonia shot?" were defined as having had a pneumococcal vaccination. We excluded hemoglobin A1c (HbA1c) monitoring from the definition of preventive care services because of a change in the question about HbA1c that made the 1997 and 2007 data incomparable.

We used SUDAAN version 9.0.1 (RTI International, Research Triangle Park, North Carolina) and SAS version 9.1 (SAS Institute Inc, Cary, North Carolina) to analyze data. We incorporated the survey sampling design and sampling weights to make results representative of the US population. We used *t* tests to assess the overall changes in rates between 1997 and 2007. In addition to testing the rate difference in multiple preventive care services, we analyzed each preventive care service to understand the rate of change in the individual service over time. We used linear regression, weighting the annual estimates by the inverse of their variances, to test for national linear trends in use of preventive care services. We considered results significant at *P* < .05 for *t* test and regression analyses. We conducted all analyses in 2008.

## Results

After adjusting for age, the overall rate of receiving all 4 preventive care services increased from 10% in 1997 to 20% in 2007 (Table). The proportion of respondents who received all 4 preventive care services increased significantly in almost all demographic subgroups. Rates increased significantly in every age group, but the largest increases were seen for people aged 65 to 74 years and those aged 75 or older. The smallest increase in any demographic category was among Hispanics (4 percentage points), and this increase did not reach significance.

The proportion of adults with diabetes who received an annual foot examination increased from 57% in 1997 to 69% in 2007, for a modeled average annual increase of 1.2 percentage points in our linear trend analysis (*P* < .001) (Figure). The proportion of adults with diabetes who received pneumococcal vaccination increased from 22% in 1997 to 39% in 2007, for a modeled average annual increase of 1.9 percentage points (*P* = .002). We observed no significant trends in receipt of annual eye examination or influenza vaccination (Figure).

**Figure.**
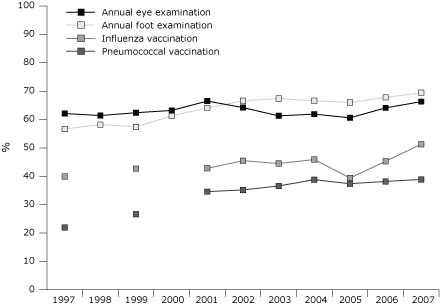
Age-adjusted rates of receiving 4 preventive care services among adults with diabetes, Behavioral Risk Factor Surveillance System, United States, 1997-2007. Data on rates of vaccination are not available for 1998 and 2000.

**Table:** 

**Service**	**%**										

	**1997**	**1998**	**1999**	**2000**	**2001**	**2002**	**2003**	**2004**	**2005**	**2006**	**2007**
Annual eye examination	62.1	61.4	62.4	63.2	66.5	64.2	61.3	61.9	60.6	64.1	66.3
Annual foot examination	56.7	58.2	57.4	61.3	64.1	66.6	67.4	66.6	66.0	67.8	69.4
Influenza vaccination	39.9	NA	42.7	NA	42.8	45.5	44.5	45.9	39.4	45.3	51.3
Pneumococcal vaccination	22.0	NA	26.6	NA	34.6	35.2	36.6	38.8	37.4	38.2	38.9

## Discussion

Only a small percentage of adults with diabetes receive all the recommended preventive care services, although this percentage is increasing. From 1997 to 2007, the rate of receiving all 4 preventive care services increased significantly overall and in every demographic subgroup, with the exception of Hispanics. When each service was examined individually, rates of receiving annual foot examinations and pneumococcal vaccinations also increased significantly, although rates of receiving annual eye examinations and influenza vaccinations did not. These observations are consistent with findings of previous studies (6-8).

Age-adjusted rate of individual preventive care practices — including annual eye examinations, annual foot examinations, influenza vaccinations, and pneumococcal vaccinations — among people with diabetes in the United States increased from 1995 to 2001 (6). Although the findings in this report show that receipt of multiple preventive care services has increased across all races and ethnicities, a lower proportion of non-Hispanic blacks and Hispanics reported receiving all 4 preventive care services compared with non-Hispanic whites. These results are consistent with a Medicare study that found that use of preventive care services, including HbA1c tests, lipid tests, eye examinations, and self-monitoring of blood glucose, by beneficiaries with diabetes increased from 1992 to 2001, and nonwhite beneficiaries consistently used fewer preventive care services and had higher rates of complications (7). Similarly, another study found that both black and Hispanic managed care enrollees were less likely than whites to use preventive care services (8). Reasons for these disparities in preventive care services are unknown; however, previous studies point to factors such as differences in providers' assumptions, beliefs, and attitudes about the patient; provider interpersonal behavior with the patient; or differences in patient-provider communication, which may lead to a less active patient who does not request preventive services during a visit (9,10).

Two previous reports have used similar methods of combining 1 or more of the ADA recommended standards of care to develop the measure of "multiple preventive care services" as a means to assess preventive care practices among adults with diabetes. Using 1994 BRFSS data, Beckles et al combined 5 of the ADA recommended preventive care measures (self-monitoring blood glucose, awareness of HbA1c, visits to a health care provider in the last year, annual foot inspection by a health care provider, and annual dilated eye examination) and found only 2.6% of insulin users reported all 5 in the previous year (11). From 2002-2004 BRFSS data, Mukhtar et al combined 3 of the ADA recommended preventive care measures (annual dilated eye examination, annual foot examination by a health care provider, and 2 or more HbA1c tests by a health care provider) and found that 39.5% of adults with diabetes reported receiving all 3 preventive care services in the previous year (5). Compared with our study and the one by Mukhtar et al, Beckles et al analyzed more preventive care services and a smaller subpopulation of adults with diabetes to asses the use of preventive care services, but all 3 reports conclude that most adults with diabetes do not meet recommendations for standards of diabetes care.

Both public and private efforts, campaigns, and interventions on the local, state, and national levels to improve preventive care services are ongoing. The Centers for Disease Control and Prevention (CDC) has implemented multiple public health strategies to improve rates of annual foot and eye examination and influenza and pneumococcal vaccination, including initiating the diabetes national objectives in 1999 (12). CDC also supports 59 state and territorial diabetes prevention and control programs that work with both private and public health partners to improve quality diabetes care. Since 1997, the National Institutes of Health and CDC have sponsored the National Diabetes Education Program, whose goal is to reduce the morbidity and mortality caused by diabetes and its complications (13). The coordinated efforts of these organizations and other state and local campaigns may have increased the use of multiple preventive care services in the last 11 years.

The findings in this report are subject to several limitations. Adults living in long-term care facilities or in households without landline telephones are not included in BRFSS surveys; thus, these results do not reflect the entire US population. The results were not adjusted for insurance status, which may have altered some of the findings, particularly for Hispanics. BRFSS data are self-reported and subject to recall bias; therefore, use of preventive care services may have been underreported or overreported. The extent to which reporting bias could affect these results is unknown. Nonetheless, validation studies have indicated that self-reported diabetes and receipt of dilated eye examinations and influenza vaccination are highly accurate (14-16).

In conclusion, although the percentage of adults with diabetes who receive preventive care services doubled from 1997 to 2007, 80% of patients did not meet ADA recommendations for continuing care, and racial/ethnic disparities persist among those who receive care. Affordability of care is often cited as an issue, but 92% of people with diabetes have some form of health insurance coverage (17). Further investigation should be undertaken to determine 1) why the rates of annual foot examination and pneumonia vaccination remain higher than rates of annual eye examination and influenza vaccination, 2) why so few adults with diabetes meet multiple preventive care recommendations, and 3) why the Hispanic population seems to receive preventive care services at a lower rate than do non-Hispanic whites and blacks. These investigations should include mixed methods — such as continued population-based surveillance of preventive care services, oversampling of Hispanic populations, and qualitative research methods — to determine the barriers to receiving preventive care, particularly among the Hispanic populations. Finally, to increase the proportion of all people with diabetes who receive multiple preventive care services, systems-level approaches, such as use of electronic medical records and patient registries, are needed that emphasize policy and environmental changes to ensure health equity and access to and delivery of quality health care.

## Figures and Tables

**Table.** Trends in Receipt of 4 Preventive Care Services (Annual Foot Examination, Annual Eye Examination, Influenza Vaccination, and Pneumococcal Vaccination) Among Adults With Diabetes, BRFSS, United States, 1997-2007^a^

Characteristic^b^	1997		2007		Percentage Point Change (95% CI)

	n	% (95% CI)	n	% (95% CI)	
**Crude total**	6,974	16 (14-17)	47,742	28 (27-29)	12 (10-14)
**Age-adjusted total**	6,974	10 (9-11)	47,742	20 (19-22)	10 (8-12)
**Age, y**					
18-44	1,053	4 (2-6)	4,026	12 (10-15)	8 (5-12)
45-64	2,767	12 (9-14)	20,870	21 (20-23)	9 (7-13)
65-74	1,920	23 (20-26)	13,074	38 (36-41)	15 (11-20)
≥75	1,234	27 (23-32)	9,772	46 (43-48)	19 (13-24)
**Sex**					
Men	2,786	10 (8-13)	19,090	21 (18-23)	11 (7-14)
Women	4,188	10 (8-11)	28,652	19 (18-21)	9 (7-12)
**Race/ethnicity**					
White, non-Hispanic	5,064	11 (10-13)	34,462	24 (21-26)	12 (9-15)
Black, non-Hispanic	928	8 (5-11)	5,808	17 (14-20)	9 (6-14)
Hispanic	672	6 (2-11)	4,000	10 (8-12)	4 (2-8)
**Education**					
Less than high school graduate	1,951	7 (5-10)	8,587	15 (12-19)	8 (4-12)
High school graduate	2,352	9 (8-12)	16,635	19 (17-21)	10 (6-12)
Some college or more	2,642	12 (10-14)	22,358	23 (20-25)	11 (8-13)
**Smoking status**					
Current smoker	1,151	7 (5-10)	7,248	20 (17-23)	13 (9-16)
Former smoker or nonsmoker	5,787	10 (9-12)	40,267	20 (18-22)	10 (8-12)
**Marital status**					
Married/cohabitating	3,506	10 (8-11)	23,892	21 (19-23)	11 (9-14)
Divorced/separated/widowed	2,893	10 (8-13)	19,730	18 (16-21)	8 (4-12)
Never married	561	9 (6-15)	3,988	19 (16-22)	10 (4-15)
**Any kind of health insurance coverage**					
Yes	6,364	11 (10-12)	26,808	21 (20-23)	10 (8-13)
No	602	2 (1-4)	2,535	12 (10-15)	10 (7-13)

Abbreviations: BRFSS, Behavioral Risk Factor Surveillance System; CI, confidence interval.

a Age-adjusted to the 2000 US standard population, except for the 4 age groups, for which crude data are presented.

b All differences from 1997 through 2007 significant at *P* < .001, except for race/ethnicity (*P* = .06).
